# The Changing Landscape of Nutrition in Cystic Fibrosis: The Emergence of Overweight and Obesity

**DOI:** 10.3390/nu14061216

**Published:** 2022-03-13

**Authors:** Julianna Bailey, Stefanie Krick, Kevin R. Fontaine

**Affiliations:** 1 Division of Pulmonary, Allergy and Critical Care Medicine, University of Alabama at Birmingham, Birmingham, AL 35294, USA; skrick@uabmc.edu; 2 Gregory Fleming James Cystic Fibrosis Research Center, University of Alabama at Birmingham, Birmingham, AL 35294, USA; 3 Department of Health Behavior, School of Public Health, University of Alabama at Birmingham, Birmingham, AL 35294, USA; kfontai1@uab.edu

**Keywords:** cystic fibrosis, nutrition, obesity, overweight, body composition, body mass index (BMI)

## Abstract

Cystic fibrosis has historically been characterized by malnutrition, and nutrition strategies have placed emphasis on weight gain due to its association with better pulmonary outcomes. As treatment for this disease has significantly improved, longevity has increased and overweight and obesity have emerged issues in this population. The effect of excess weight and adiposity on CF clinical outcomes is unknown but may produce similar health consequences and obesity-related diseases as those observed in the general population. This review examines the prevalence of overweight and obesity in CF, the medical and psychological impact, as well as the existing evidence for treatment in the general population and how this may be applied to people with CF. Clinicians should partner with individuals with CF and their families to provide a personalized, interdisciplinary approach that includes dietary modification, physical activity, and behavioral intervention. Additional research is needed to identify the optimal strategies for preventing and addressing overweight and obesity in CF.

## 1. Introduction

Cystic Fibrosis (CF) is a rare, life-shortening multi-system organ disease that affects 30,000 people in the United States and 70,000 people worldwide [1]. Pulmonary failure is the main cause of death in this population; the heavy involvement of the gastrointestinal system creates significant nutritional impairments [2]. CF was initially called “Cystic fibrosis of the pancreas” due to the aggressive involvement of the GI system [3]. Historically, malnutrition and underweight have been the prevailing nutritional issue in people with CF (PwCF). Nutritional status has long been defined by body mass index (BMI) in this patient population due to epidemiological evidence that BMI is closely correlated with lung function and, ultimately, survival. Due to the positive correlation between BMI and lung function, the Cystic Fibrosis Foundation (CFF) has set BMI goals of at or above the 50th percentile for children and 22 kg/m^2^ or higher for adult females and 23 kg/m^2^ or higher for adult males with CF [4]. For this reason, and due to the history of malnutrition with CF, most nutrition interventions have focused on increasing BMI. A high-calorie, high-protein, high-fat diet is recommended for most PwCF, along with oral supplements and sometimes supplemental enteral feeds to help patients achieve the BMI goals associated with the best health outcomes [5]. Aggressive nutrition support has been recommended in pediatric patients with CF to avoid/prevent malnutrition, and to promote catch up growth. Moreover, adequate nutritional status has been associated with reduced pulmonary exacerbations and improved lung function [6].

However, overweight and obesity have emerged as an important issue in the CF population due to advancements in therapy and increased longevity, especially in recent years with the introduction of CF transmembrane conductance regulator (CFTR) modulator therapies [7,8,9,10]. There are limited data on overweight and obesity in CF, and few studies related to the impact of obesity on clinical outcomes in CF. The purpose of this review is to critically examine the literature on overweight and obesity in CF, and to make recommendations for clinical practice and future research in this area.

## 2. Overweight and Obesity in Cystic Fibrosis

BMI has been used to define overweight and obesity, with overweight classified as a BMI 25–29.9 kg/m^2^ and obesity as BMI of ≥30 kg/m^2^ in adults. In children, BMI percentiles are used to measure growth and 85–95th percentile indicates overweight with >95th percentile indicating obesity in children [11]. In the United States, 42.2% of adults are obese, with 9% being severely obese [12]. The prevalence of childhood obesity in the U.S. is 19.3% [13]. Obesity is associated with the development of many chronic and potentially life-threatening health conditions such as heart disease, cancer, and diabetes and virtually every organ system can be adversely affected by obesity. The medical cost associated with obesity is estimated to be USD 149 billion, making it both a serious public health crisis and economic issue in the general population [14]. While malnutrition has been the primary issue in CF due to increased work of breathing and malabsorption, obesity and overweight are becoming a growing concern [15]. Dyslipidemia and insulin resistance have been observed in the CF population; it is unknown how these factors affect risk for development of heart disease and diabetes, and how obesity may affect risk for development of comorbidities in CF due to the life-shortening nature of this disease [16,17,18].

Nutritional status has largely been defined by BMI in the CF population due to associations between BMI and pulmonary disease. Given the changing landscape of nutritional status in PwCF and the rise in overweight and obesity, body composition as a predictor of pulmonary and clinical outcomes has also become an area of interest. Further examination of body composition is merited given that the World Health Organization defines obesity as “an abnormal or excessive accumulation of fat that poses a risk to health” [19]. Research on obesity and body composition in the general population has demonstrated that body fat distribution, particularly increased upper body fat, visceral fat, and intramuscular fat, are important predictors of metabolic consequences commonly associated with obesity [20]. Evidence suggests that PwCF tend to have lower fat-free mass than healthy controls [21,22,23,24,25,26,27] and that lower fat-free mass is associated with lower lung function in CF [21,23,28,29,30,31]. Other body composition abnormalities including ectopic fat deposition with fatty replacement of the pancreas, central adiposity, visceral adiposity, and normal weight obesity have been observed in CF [28,29,32,33,34,35]. Few studies have explored the implications of increased abdominal fat and visceral fat specific to CF; however, one cross-sectional study of adults with CF found that high levels of visceral fat in CF were associated with decreased insulin sensitivity [36]. While associations between some clinical outcomes and body composition exist in the literature, more studies are needed to ascertain the role of body composition in nutritional assessment, and other clinical aspects of CF treatment including the management of overweight and obesity.

### 2.1. Prevalence

Several studies have assessed the prevalence of overweight and obesity in CF. A CF center-specific cross-sectional study in children at the Pittsburg CF Center found an increased rate of overweight and obesity in children with CF, with 15% of children qualifying as overweight and 8% of children were obese, based on BMI percentile [15]. Interestingly, 50% of the overweight children and 20% of the obese children suffered from malabsorption caused by CF-associated exocrine pancreatic insufficiency [15]. A recent single center study conducted in the Minnesota adult CF center found that >30% of patients were overweight or obese between the years of 2015–2017 [37]. A longitudinal analysis of the U.S. CF Foundation Patient Registry indicates that the prevalence of overweight and obesity increased over the past 20 years in both children and adults with CF. Between the years of 1998 and 2017, the percentage of overweight increased from 7% to 16% and the proportion of obese patients increased from 2% to 6%, which was noted to be a 345% increase in the number of PwCF who are obese due to CF population increase in the registry [38]. Increased overweight and obesity has also been observed in the UK CF population, and in a CF genotype typically associated with pancreatic insufficiency. An analysis of UK CF registry data for the year of 2002 attempted to determine the prevalence of overweight and obesity in children and adults with the most common CF mutation, DeltaF508 homozygous. Results indicated that 9% of PwCF and the DeltaF508 homozygous genetic mutation were overweight and 1% were obese [39]. While rates seem low compared to the obesity prevalence in the general population, this is significant because overweight and obesity are not expected in patients who are homozygous for DeltaF508 as this genotype has historically been associated with more severe nutritional deficiencies [40]. A cross-sectional observational study of 68 adults and children with CF in a Greek CF center found that 13.2% of patients with CF were either overweight or obese [35]. Analysis of a single CF center in Spain found that 6% of children with CF were overweight and 1% were obese, and that overweight/obese status did not provide improved pulmonary function [18]. Likewise, in Italy, a single site study of adults with CF found that 22% were either overweight or obese [41]. In addition to cross-sectional studies found in the literature, a longitudinal cohort study in Toronto of 909 adults with CF and that found that the percentage of overweight and obese CF patients increased from 7% to 18.4% between 1985 and 2011, similar to trends observed in the U.S. CF Patient Registry [8,38]. Prevalence of overweight and obesity in CF from cross-sectional and longitudinal studies in international CF samples is presented in Table 1. Given early data from weight gain on new CFTR modulators that are available to 90% of the CF population, we can expect these numbers to rise even further [9,42,43,44].

### 2.2. Etiology

#### 2.2.1. Genetics

Multiple factors may contribute to the decreasing proportion of malnutrition and increasing prevalence of overweight and obesity in CF. There is evidence to suggest that some people are genetically predisposed to development of overweight and obesity [46]. In the CF population, some studies have found that obesity occurs more frequently in patients with less severe genetic mutations and patients who are pancreatic sufficient [8,39]. A US CF Registry-based study conducted by Flume and colleagues found that the prevalence of obesity was higher among individuals with CF who had class IV or class V CF causing genetic mutations, which are typically less severe mutations. However, a recent single CF center study in Minnesota found that overweight and obesity were present even in patients with genotypes associated with more severe disease [37], and a UK CF Registry-based study found that overweight and obesity were present in patients homozygous for F508del, which is known to present a more severe phenotype and is associated with pancreatic insufficiency [39].

#### 2.2.2. Dietary Causes

The unrestricted high-calorie, high-fat diet that is often called the “legacy CF diet” likely contributes to positive energy balance that promotes excessive weight gain. PwCF have long been advised and behaviorally conditioned to consume an unrestricted high-calorie diet with an emphasis on weight gain since childhood and have received vastly different messaging around nutrition than the general population [4]. Indeed, studies of diet quality in children with CF have shown intake high in saturated fat, trans fat, and total calories but low in nutrient-dense foods such as fruits and vegetables [47]. A similar pattern has been observed in adults with CF in a cross-sectional study that demonstrated intake high in added sugars, refined grains, trans-fatty acids, and low intakes of whole grains and dietary fiber [36]. Further, poor diet quality was associated with high levels of visceral adipose tissue in this study [36]. Until recently, CF nutrition guidelines recommended an unrestricted high-calorie diet, with little focus on nutrient density or diet quality. As clinical care and treatments for CF have improved over the past decade, the high-calorie CF diet may have had led to positive energy balance and excessive weight gain in a subset of PwCF. Extended life expectancy is prompting a focus on diet quality to promote optimal health outcomes and prevention of other chronic lifestyle diseases in CF.

#### 2.2.3. CFTR Modulator Therapy

Highly effective drugs that treat CF at the cellular level, known as CFTR modulators, are now available for up to 90% of the population with CF. CFTR modulators have been shown to improve pulmonary function, quality of life, and in some cases, cause weight gain and increase BMI. A recent systematic review concluded that the effect of CFTR modulators on anthropometric measurements is dependent on genetic mutation and modulator formulation [48]. Ivacaftor, the first CFTR modulator approved, was available to roughly 6% of the CF population who have gating mutations and was shown to increase weight and linear growth in children with CF [49,50,51]. In a long-term study of the clinical effects of ivacaftor, Guimbellot et al. observed that the proportion of overweight increased from 16% to 25% in adults and from 9% to 18% in pediatric patients over 5.5 years on the drug [10]. The rate of obesity remained stable in pediatric patients but increased slightly from 8% to 11% in adults [10]. A new highly effective CFTR modulator, elexacaftor-tezacaftor-ivacaftor (ETI), is now available to up to 90% of the CF population and has been shown to increase BMI in adolescents and adults with CF by 0.9–1.1 kg/m^2^ in phase III clinical trials [43,44]. A single-center retrospective study recently confirmed weight gain in adults on ETI in the real-world setting, with an average BMI increase of 1.5 kg/m^2^ after 12 months on ETI [9].

Few studies have explored mechanisms of weight gain on CFTR modulators, but decreased resting energy expenditure, increased caloric intake, and improved intestinal absorption are postulated to play a role in adults with gating mutations taking ivacaftor only [52]. One study of dietary changes on ivacaftor demonstrated that American participants increased their fat intake significantly, while Italian participants increased both total calorie and fat intake while taking ivacaftor [53]. This may be due to drug package instructions that advise patients to take the drug with “a fat containing food” twice per day while the quantity of fat that should be consumed for optimal drug efficacy is unknown. Some evidence also indicates that children with CF who took ivacaftor showed improvement, and in some cases, reversal of exocrine pancreatic insufficiency [54,55,56]. Improved pancreatic function likely plays a role in weight gain in children who take ivacaftor.

It is important to understand whether weight gain on CFTR modulators is due to fat mass or lean body mass accrual. There are little data on body composition changes on CFTR modulators, but one study of adults who took ivacaftor found an increase in both fat mass and fat-free mass as measured by DEXA after 3 months on the drug [52]. Another study found an increase in fat mass using bioelectrical impedance analysis after 28 days on ivacaftor [57]. In a long-term open label extension study of ivacaftor, King et al. found that both weight and fat mass significantly increased after 6 months of treatment and, at two years on the drug, 64% of weight gained was fat mass. Additionally, 25% of participants were classified as overweight and 10% were obese by the end of the two-year study [58]. More studies of longer duration are needed to examine body composition and fat distribution changes on CFTR modulators in the setting of unintended weight gain and the development of overweight and obesity on these drugs.

#### 2.2.4. Social Determinants of Health

Social determinants of health are defined as environmental conditions that affect health and quality of life outcomes and risks [59]. Limited access to healthy food correlates with other unmet needs that indicate poverty. Poverty and food insecurity are both associated with obesity in the general population [60,61]. Health disparities related to poverty or low socioeconomic status (SES) have been documented in CF and affect survival, CF-related clinical outcomes, and access to optimal healthcare for CF [62,63,64]. Low SES is associated with obesity in the general population but socioeconomic factor contributions to overweight and obesity in the CF population have not been explored. Food insecurity and limited access to healthy food could increase risk for overweight and obesity in the population due to reliance on high-calorie convenience foods that lack nutrient density. Additionally, PwCF spend an average of 2–3 h per day on a complex medical treatment regimen, which leaves little time for healthy meal planning, particularly in the setting of food insecurity that limits access to healthy food. It is reasonable to suspect that a similar association between obesity and poverty and food insecurity observed in the general population may exist in CF, but national CF Registry-based studies are needed to understand the role of social determinants of health in the development of overweight and obesity in the CF population. Additional research could provide data to create targeted interventions to prevent over-nutrition in subsets of the CF population experiencing socioeconomic hardship.

## 3. Consequences of Overweight and Obesity in Cystic Fibrosis

### 3.1. Lung Function

There is a strong positive association between BMI and lung function in the CF population [2]. A large Canadian CF Registry analysis found that while overweight and obesity were associated with an increase in lung function, the magnitude of the increase was significantly less in the obese group than in the adequate-weight reference group [8]. Emerging evidence suggests that overweight and obesity may not provide benefit with respect to pulmonary function and could even be detrimental. In a single-center cross-sectional study conducted by Hanna et al., no associations were observed with overweight/obese status and lung function, prompting the authors to conclude that overweight and/or obesity does not confer pulmonary benefits in CF [15]. Several other studies examining prevalence and outcomes of obesity in CF have found that overweight and obesity were not protective with respect to lung function [8,15,18,65]. Additionally, emerging evidence suggests that in children with CF who are pancreatic sufficient, a BMI > 85th percentile has a detrimental effect on pulmonary function [66]. Large national registry-based studies are needed to determine longitudinal associations between higher BMI and pulmonary outcomes in the CF population.

### 3.2. Cardiometabolic Parameters

Few studies have addressed the effect of obesity on other comorbidities in CF. Shorter life expectancy has typically not allowed for patients with CF to live to the age where obesity-associated comorbidities such as heart disease, cancer, and type 2 diabetes would emerge. Additionally, at least one study estimates that up to 50% of adults with CF develop CF-related diabetes, which is a unique disease entity distinct from both type 1 and type 2 diabetes, but with features of both insulin deficiency and insulin resistance [16]. The endocrine abnormalities inherent to CF make understanding the association between obesity and development of diabetes more difficult to assess in this population. However, a cross-sectional study by Coderre and colleagues found that 13% of adults with CF had a BMI > 25 kg/m^2^ and these overweight/obese patients had higher fasting insulin, total cholesterol, and LDL cholesterol than patients with CF at lower BMI. They also found that LDL cholesterol and insulin area under the curve were associated with lower lung function [67]. In a comparison of underweight, normal weight, and overweight PwCF to determine differences in insulin levels, insulin response, and prevalence of diabetes, 5.5% of patients were overweight and overweight patients had higher fasting insulin, higher insulin resistance (HOMA-IR), and higher insulin levels during an oral glucose tolerance test [65]. Authors concluded that overweight patients with CF may be at increased risk for the development of diabetes [65]. Dyslipidemia has also been observed in the CF population and studies have found a positive association between total cholesterol and triglyceride levels and BMI in the CF population [8,67,68,69,70]. In a recent study, ETI was shown to increase total cholesterol, HDL, and LDL in adult patients with CF-related diabetes. The implications of dyslipidemia in PwCF are not clear, and the role of dietary intervention in CF dyslipidemia is unknown [9]. More longitudinal research is needed to understand the association between overweight and obesity in CF and how this affects risk for the development of diabetes and cardiovascular disease.

### 3.3. Lung Transplantation

Lung transplantation is a treatment option for end-stage pulmonary diseases including CF. Large database evidence suggests that both malnutrition and overweight are associated with increased risk of death following a lung transplant. Currently, class II or III obesity (BMI ≥ 35 kg/m^2^) is considered an absolute contraindication to lung transplant, and class I obesity (BMI 30–34.9 kg/m^2^) is considered a relative contraindication. In a recent study of 546 lung transplant patients, patients who were overweight or obese had a significantly higher mortality rate. Of note, only two of the patients who had CF were overweight and none were obese. More research is needed to understand how overweight and obesity affect lung transplant outcomes in CF, and assessing body fat distribution may be especially important as abdominal and visceral fat can play a role in restrictive lung disease [71].

### 3.4. Weight Stigma and Psychological Consequences

Obesity is common in the general population but is a highly stigmatized condition in many settings including the medical environment. Evidence suggests that people with obesity experience prejudice, derogatory comments, and that in some cases, health care professionals hold negative perceptions of patients who are overweight or obese. Patient experience of weight stigma can also lead to increased stress and subsequent avoidance of clinical care [72]. A recent systematic review of the impact of weight stigma in overweight and obese individuals found that weight stigma was positively associated with cortisol levels, diabetes risk, eating disturbances, depression, anxiety, body image issues, and was negatively associated with self-esteem [73]. In the general population, weight stigma has been associated with decreased motivation to change eating patterns and less healthy eating behaviors, which could lead to negative health consequences [74]. While there are no studies related to weight stigma in overweight and obese PwCF, there is a growing body of evidence related to body image disturbances and even eating disorders in this population [75,76,77]. Clinicians should therefore take a sensitive and empathetic approach when having discussions about overweight and obesity, and be cognizant of the psychological consequences of weight stigma. Additionally, it would be beneficial to include the CF team social worker and/or psychologist early on in the care of these patients to address the mental health impact of overweight and obesity.

## 4. Treatment Strategies

Management of overweight and obesity in the general population typically involves weight loss given evidence that a reduction of 5–10% body weight can reduce the risk of cardiovascular disease, improve lipid panels, decrease blood pressure, and decrease risk of diabetes [78,79]. Even a sustained weight reduction of as little as 3–5% has been associated with some positive health benefits and reduced health risk [80]. Weight loss in overweight and obesity can be effectively achieved by creating negative energy balance through dietary modification to reduce energy intake paired with physical activity to increase energy expenditure, and supported by behavioral counseling to facilitate lifestyle change [81,82]. Studies show that this approach can produce a 3–5% body weight loss that is associated with an improvement in health parameters [83]. Other markers of health can include cardiometabolic parameters such as lipid panels, blood pressure, diet quality, eating behaviors, hemoglobin A1c (in diabetic patients), body composition parameters, and quality of life. When lifestyle modification is unsuccessful, medication and even surgical management may be necessary.

To our knowledge, no studies exist regarding treatment strategies for overweight and obesity in in CF. Studies conducted thus far suggest some associations between overweight/obesity and comorbidities in CF, worse lung transplant outcomes, dyslipidemia, and increased diabetes risk with higher fasting insulin levels. Therefore, further work is needed to better understand the etiology, consequences, and treatment and prevention strategies for PwCF who are overweight and obese. It is important to have a full evaluation by all members of the multidisciplinary CF care team when a patient has unintended weight gain that results in overweight or obesity. Causes of weight gain such as medications and other disease processes should be ruled out before pursuing interventions for overweight and obesity management. While research is conducted to identify the optimal approach to overweight and obesity in CF, it is reasonable to utilize evidence-based methods for the general population presented in this section.

### 4.1. Dietary Intake Recommendations

The CF Foundation has not formally updated CF Nutrition Guidelines since obesity and overweight emerged as a significant issue or since highly effective CFTR modulators recently became available for most of the CF population. However, several organizations have created evidence-based guidelines in recent years, which are presented in Table 2. Most recently, the Academy of Nutrition and Dietetics (AND) published guidance based on a systematic review that suggests that there is no evidence to support that PwCF have any benefit from consuming a dietary pattern outside of what is recommended for the general population [84]. This guideline also emphasizes just as some patients with CF experience under-nutrition, some patients may require caloric reduction with a focus on improving diet quality given that undesired weight gain, overweight, and obesity have been reported in a subset of the CF population, especially with CFTR modulator use. Both the Australia and New Zealand CF Nutrition Guideline and the AND CF Nutrition Guideline emphasize taking an individualized approach to nutrition care based on patients’ genetic mutations, clinical status, laboratory values, personal health goals, nutrition status, culture, and food preferences [84,85]. The AND guideline suggests a diet rich in whole grains, fruits, vegetables, seafood, legumes, lean protein, nuts and beans, and low-fat dairy as these foods have been associated with positive health outcomes in the general population [84].

In the general population, a deficit of 500 calories per day can produce weight loss of one pound per week [87]. Negative energy balance may be achieved through diet alone or through a combination of diet and exercise. Evidence suggests that a variety of dietary patterns can be effective for calorie reduction including low carbohydrate, low fat, portion control, and the Mediterranean diet pattern with calorie restriction [81,82,88]. Other dietary trends for weight loss are gaining popularity in the general population, including time-restricted plans such as intermittent fasting; however, there is currently no evidence to suggest efficacy in the CF population. Given that the best predictor of weight loss is adherence to a chosen dietary plan, it is important to select the diet best suited for the individual in order to promote the sustainability of dietary patterns and lifestyle changes [89]. PwCF have been counseled to eat a high-calorie diet since early childhood, so clinicians should recognize that shifting a dietary intake will take time and often trial and error to find the pattern that works best for each individual. Small incremental changes may be useful to build confidence and avoid overwhelming patients [90]. Table 3 presents suggestions for modifications to the legacy CF diet adapted from the Academy of Nutrition and Dietetics Evidence Based Guideline for Nutrition in Cystic Fibrosis and the Dietary Guidelines for Americans with the goal of reducing calories and increasing nutrient dense foods and foods known to promote health and reduce the risk of chronic disease.

In considering dietary change recommendations for PwCF as overweight and obesity emerge, it should be noted that malnutrition still exists in subsets of the CF population, particularly in those who are not eligible to take CFTR modulators due to their genetic mutations and in patients with advanced lung disease. PwCF who also experience malnutrition will likely continue to require a nutrient-dense high-calorie diet to optimize nutrition status. The broad spectrum of nutrition status observed in CF currently highlights the need for highly individualized nutrition therapy. The CF Care Team Registered Dietitian should work carefully with patients in co-producing customized nutrition care plans to help patients meet personal goals and optimize health outcomes, based on individual clinical status, cultural considerations, and food preferences. Emphasis should also be placed on screening for food insecurity and other social determinants of health that could impact patient’s ability to afford healthy foods. Registered Dietitians can assist patients in planning healthful meals and eating patterns that are affordable and should work in tandem with the CF Social Worker and other care team members to provide resources to improve food access for patients experiencing food insecurity.

### 4.2. Physical Activity

While diet is a crucial element in the management of overweight and obesity, a multidisciplinary lifestyle intervention is recommended [91]. Exercise is an important component of comprehensive lifestyle plans to treat overweight, obesity, and associated comorbidities. Regular physical activity has been shown to assist in creating negative energy balance for weight loss and improve cardiometabolic risk factors [92]. In the general population, it is recommended to participate in 150 min per week of moderate physical activity or 75 min per week of vigorous activity. To promote weight loss in the absence of dietary changes, 225–420 min of exercise is recommended. However, physical activity paired with reduced caloric intake is recommended as this strategy is more likely to promote weight loss [93]. Research has also consistently demonstrated that physical activity is necessary for weight loss maintenance in the general population [80]. Aside from weight loss, regular physical activity is associated with reduced risk of cardiovascular disease and diabetes in obese individuals, independent of weight loss [92]. In the CF population, physical activity, particularly strength training, is an important component of exercise plans in order to promote preservation and accrual of lean body mass [94], which is associated with improved pulmonary function in this population. Additionally, exercise in CF also provides additional benefits beyond weight management, including increased aerobic and pulmonary capacity and airway clearance [95]. Further, exercise can provide mental health benefits that could combat anxiety and depression associated with overweight and obesity [96]. The CF Care Team Physical Therapist is an invaluable resource in creating customized exercise plans to help patients meet their personal health, weight, and body composition goals and that synergize with the nutrition care plans provided by Registered Dietitians.

### 4.3. Behavioral Interventions

Comprehensive lifestyle interventions for overweight and obesity include dietary intervention, physical activity, and behavioral approaches to lifestyle change [97]. A systematic review that defined successful weight loss interventions for obesity as ≥5% initial body weight loss maintained at 12 months found that 92% of successful interventions included a behavioral component [82]. Intensive behavioral therapy (IBT) for obesity that includes nutritional guidance and exercise has been shown to produce significant weight loss, lower blood glucose levels, decrease waist circumference, and decrease blood pressure. Additionally, IBT decreased the development of diabetes by 50% in patients who had elevated blood glucose at baseline [98]. Many behavioral change theories, models, and strategies provide an evidence-based approach to changing behaviors related to energy balance in the treatment of overweight and obesity. Cognitive behavioral therapy (CBT) and motivational interviewing (MI) are two modalities of behavioral change that have been used with success in obesity treatment [81,99]. CBT involves such skills as self-monitoring, goal setting, problem solving, and stimulus control [81]. MI is an approach that emphasizes partnership for a client-driven lifestyle change intervention [100]. Many different behavior change models exist and a variety of combinations of behavioral techniques and strategies can be used to facilitate behavior change.

The intensity of the comprehensive lifestyle intervention seems to have an impact on the efficacy of behavioral interventions for the management of overweight and obesity. Research has shown that 14 sessions in a 6-month time frame led to more weight loss than a less frequent intervention of 12 or less sessions in 6 months [97]. It should be noted that most patients with CF see their care team in the clinic once every 3 months, and that more frequent multidisciplinary visits that provide comprehensive lifestyle intervention-focused on management of overweight and obesity may be warranted. Telehealth and home-based interdisciplinary lifestyle interventions may be an option for increasing frequency of visits centered around treatment of overweight and obesity, particularly for patients who live far away from their CF care centers.

Successful behavioral interventions targeting BMI in children with CF have been conducted, although these interventions are typically focused on normalizing growth and promoting weight gain. These interventions in the pediatric CF population have involved, with strong parental support and participation, implementing a behavioral intervention in combination with nutrition education, and this has been found to be more effective than nutrition education alone [101]. These behavioral nutrition interventions focus on positive reinforcement and promoting positive mealtime behaviors to prevent growth decline in children with CF. It is possible that these behavioral intervention strategies could potentially be used to prevent the development of overweight and obesity in children with CF.

Only one behavioral nutrition intervention conducted in adults with CF was identified in the review of the literature. This randomized controlled trial was a Social Cognitive Theory home-based behavioral intervention called “Eat Well with CF” and had a duration of 10 weeks. While this intervention was successful in improving self-efficacy scores related to nutritional self-management, there was no significant change in BMI or quality of life at the end of the intervention [102]. Self-efficacy is believed to be crucial for facilitating and maintaining behavioral changes [100]. While this intervention did not produce a significant change in BMI, it is possible that the improvement in self-efficacy could be an early indicator of behavioral change, especially given the short duration of the intervention [102]. It is also possible that early changes in body composition occurred but were not captured by measuring BMI only. Home-based behavioral interventions that involve both nutrition and physical activity components could be an avenue for increasing the frequency and intensity of multidisciplinary weight management interventions, especially given that telehealth is growing in popularity, with several studies documenting a high level of patient satisfaction and positive experience of telehealth care in CF [103,104].

### 4.4. Weight-Neutral Approaches

While weight loss is the most common goal in the treatment of overweight and obesity due to associations with improved cardiometabolic outcomes, it is unknown if the benefits associated with a 5–10% body weight loss in the general population translate to PwCF. Given that normal weight obesity and lean body mass depletion have been documented in CF [29], it is unclear if weight loss interventions for overweight and obese individuals with CF will result in predominantly fat loss and could cause further reduction in lean body mass stores. Body image issues and eating disorders have been documented in CF, and this population deals with an intense focus on weight patterns and BMI throughout their lifespan, due to the association between BMI and pulmonary function and guideline recommendations based on BMI and growth [77]. There is also some evidence that suggests that the influence of eating experiences in childhood are sustained into adulthood in CF [99]. For these reasons, some individuals may instead prefer to focus on other markers of health as outcomes, rather than weight, when designing a plan to address overweight or obesity and related health consequences. The Health at Every Size^®^ (HAES^®^) approach is a weight-inclusive model that focuses on improving physical, behavioral, and psychological parameters rather than promoting weight loss. This method also places emphasis on body acceptance and improving the individual’s relationship with food [105,106]. HAES^®^ has been gaining popularity in the general population and has been studied regarding overweight and obesity. A recent systematic review of randomized controlled trials in overweight and obese individuals found that the HAES^®^ approach is associated with improved quality of life, improved cardiovascular endpoints, increased physical activity, reduction in disordered eating, reduction in binging as well as improved diet quality [107]. Despite the weight-neutral approach utilized, some studies have demonstrated reduction in BMI, waist circumference, and fat loss as a result of the HAES^®^ interventions [108]. While there are no data on the efficacy of HAES^®^ in CF, clinicians should be aware of this approach as individuals with CF who have a history of body image disturbances or disordered eating may prefer a weight-neutral approach to addressing the health consequences associated with the development of overweight and obesity. More research is needed on comprehensive health behavior interventions, including weight-neutral approaches to improve cardiometabolic outcomes and quality of life in the CF population.

### 4.5. Medical and Surgical Treatment

When lifestyle approaches are ineffective, pharmacological approaches are recommended to treat overweight and obesity in the general population when BMI is >30 kg/m^2^ or >27 kg/m^2^ with comorbid conditions in adults [109]. Several drugs are approved by the FDA to treat obesity and include orlistat, lorcaserin, liraglutide, topiramate/phentermine, naltrexone/bupropion, and newly approved semaglutide [110]. In addition, prescription appetite suppressants are available and include phentermine, benzphetamine, diethylpropion, and phendimetrazine. It should be noted that appetite suppressants are only approved for short-term use of 12 weeks or less in the general population. Orlistat, Liraglutide, and setmelanotide are the only drugs approved for use in adolescents. However, setmelanotide is only approved for three specific rare genetic conditions which do not include CF. None of these drugs have been studied in CF and their use should be carefully considered by the medical team, weighing the risks and benefits with patients and their families. Of note, use of Orlistat may not be ideal in PwCF who also have pancreatic insufficiency as this could compound malabsorption and lead to vitamin and essential fatty deficiencies in this population that is already at high risk of micronutrient deficiencies.

A variety of bariatric procedures are available for the management of obesity and include surgical (gastric bypass, gastric banding, sleeve gastrectomy) and non-surgical (intragastric-balloon) options. Bariatric procedures are considered in the general population when BMI is ≥40 kg/m^2^ or BMI ≥ 35 kg/m^2^ with one or more severe obesity-related complications that can be improved with weight loss [109]. There currently is no evidence on outcomes regarding bariatric procedures to treat obesity in CF, and this could lead to micronutrient deficiencies and worsened gastrointestinal issues, particularly in patients who have exocrine pancreatic insufficiency. Given what is known about nutrition risk in the CF population, bariatric surgery may not be an ideal option for PwCF until more evidence is available.

## 5. Discussion

Nutrition status as measured by BMI is closely linked with pulmonary outcomes and survival in CF [4]. As life expectancy continues to increase in this population with the use of CFTR modulators, non-traditional nutrition issues have emerged, and overweight/obesity have become areas of interest in CF clinical care and research. Studies indicate that over-nutrition is increasing in CF, and this has been linked to insulin resistance and other hormonal disturbances, which are postulated to play a role in development of diabetes and the trend of increased central fat distribution observed in this population [65,67]. Additionally, central adiposity, visceral adiposity, and normal weight obesity have been documented in CF and could have negative health consequences [28,33,34,35].

Additional perspective is needed to determine if excess weight and adipose tissue distribution in CF are causally related to pulmonary and cardiometabolic outcomes. Specifically, additional studies are needed to determine the interplay between central adiposity and CF-related endocrine, metabolic, and pulmonary outcomes. Given the association between fat-free mass and lung function in CF, future research should also focus on exploration of the causes and predictors of decreased fat-free mass so that interventions can be developed to promote accrual of lean body mass and reduction in central adiposity, regardless of BMI status, in PwCF. While weight loss is a common goal of obesity intervention success, clinicians should also track body composition changes to ensure that lean body mass is preserved and optimized during weight reduction.

The care team should be cognizant of the stigma associated with overweight and obesity and the psychological impact of undesired weight gain. It should be noted that some individuals with CF may prefer weight-neutral interventions that place emphasis on health-promoting behaviors and repairing their relationship with food rather than weight loss as their main goal. Collaborating with the CF care team Social Worker and/or Psychologist is advised, both for support around these issues and assistance with behavioral strategies to promote optimal health and wellbeing during weight management interventions.

There is limited evidence on the optimal diet for PwCF, especially in the setting of new highly effective CFTR modulators and the development of overweight and obesity in this population. Caloric deficit is necessary for weight loss, and many dietary approaches have been shown to promote weight reduction in the general population. The sustainability of any intervention relies on an individual’s ability to adhere to the chosen regimen. It is therefore important to take an individualized approach to nutrition care and weight management plans, and it is the role of the CF care team Registered Dietitian to partner closely with PwCF and their families to determine a customized nutrition care plan that is the best fit for patient preferences, culture, and clinical status. Registered Dietitians should also closely collaborate with the CF team Physical Therapist and Social Worker/Psychologist to create a comprehensive lifestyle approach to address overweight and obesity that works for each patient’s individual needs. When these comprehensive lifestyle interventions are unsuccessful, medical management of obesity may be considered in overweight and obese individuals with CF. Given the paucity of evidence regarding weight loss drugs and bariatric surgery in CF, clinicians should carefully weigh the risks and benefits of medical management considering patient and family goals and only pursue those interventions in highly specialized centers.

Additional research is needed to determine the optimal dietary pattern for PwCF, particularly in the setting of overweight and obesity. Research should also explore the efficacy of different dietary patterns as well as weight-neutral approaches in improving cardiometabolic outcomes independent of weight loss in PwCF who are overweight or obese. Qualitative studies that explore patient and family attitudes and perceptions of their weight status and body composition, as well as what would make effective weight management interventions in CF, are also warranted. Using patients and family voices combined with emerging scientific evidence to guide interdisciplinary behavioral nutrition interventions for weight management is recommended.

## 6. Conclusions

A comprehensive interdisciplinary approach to lifestyle change, including nutrition care plans, enjoyable customized exercise, and behavioral strategies, is necessary to address the new challenge of overweight and obesity in CF. The CF Care Team should take a sensitive, highly individualized approach to enhance the sustainability of lifestyle interventions to address overweight and obesity, as well as partnership in care. Expanding the research on overweight and obesity in CF is necessary to determine the impact on cardiometabolic, pulmonary, and quality of life outcomes, and to determine optimal behavioral nutrition interventions for clinical practice as longevity continues to increase in this population.

## Figures and Tables

**Table 1:** Studies examining prevalence of overweight and obesity in CF.

Reference	Population	*N*	% Overweight	% Obese
Flume et al., 2019 [38]	United States CF Registry Data, Age ≥ 2 years	42,988 across study duration from 1998–2017	16% in 2017	6% in 2017
González Jiménez et al., 2017 [18]	Spain, 12 hospitals Age 4–57 years	451	6%	1%
Gramegna et al., 2021 [41]	Italian, multi-center Adults Age 32–45 years	321	20%	2%
Hanna et al., 2015 [15]	United States, single CF Center Children Age 2–18	226	15%	8%
Harindhanavudhi et al., 2020 [37]	United States, single CF Center, Minnesota Adults	484	25.6%	6.6%
Kastner-Cole et al., 2005 [39]	United Kingdom CF Registry Data Adults and children with F508del mutation	1869 children 1181 adults	9% in adults and children	1% in adults and children
Panagopoulou et al., 2014 [35]	Greece, single CF Center Age 2–38 years	68	6%	7%
Stephenson et al., 2013 [8]	Toronto single CF Center Adults	651	18.4%	3.3%
White et al., 2015 [45]	Australia, children from 16 inpatient hospitals	832	8.8%	9.9%

**Table 2:** Summary of cystic fibrosis nutrition guidelines.

Guideline Reference	Total Calorie Intake	Macro-Nutrient Balance	Overweight/Obesity
Cystic Fibrosis Foundation, 2008 [4]	110–120% of estimated energy requirements for general population	35–40% of calories from fat 40–45% of calories from carb 20% of calories from protein	N/A
ESPGAN, 2016 [86]	110–120% of energy requirements for same age healthy children and adults	20% of calories from protein; notes lack of evidence for recommending macro-balance	“We suggest adjusting energy intake upward to achieve normal growth and nutritional status while avoiding obesity” [86]
Thoracic Society of Australia New Zealand, 2018 [85]	110–200% of the population-based energy requirements emphasizing frequent RD assessment and individualized energy intake goals	Upper limit of 25% of calories from protein	High BMI in Pediatrics: Overweight: BMI 85th to <95th BMI Obese: >95th percentile High BMI in Adults: ≥27 kg/m^2^ AND/OR unintentional weight gain from previously acceptable BMI of >5 kg within a year [85]
Academy of Nutrition and Dietetics CF Nutrition Guideline, 2020 [84]	110–200% of the population-based energy requirements emphasizing RD frequent assessment and individualized energy intake goals	“Macronutrients in same percentage distribution as is recommended for the typical, age-matched population”	“For individuals with CF who are overweight or obese, it is reasonable for the RDN or international equivalent to advise an age-appropriate diet that emphasizes foods associated with positive health outcomes in the general population, with energy needs adjusted to achieve or maintain normal growth (pediatrics) or BMI status (adults).” [84]

**Table 3:** Dietary modifications for a generally healthful eating pattern.

Food Group	Recommended Foods	Foods to Consider Reducing
Carbohydrates	Whole grains (at least half of grains should be whole grains). Whole wheat bread, buns, rolls, tortillas, and crackers Whole wheat pasta Brown rice Wild rice Quinoa Oats Barley Whole grain cereals	Refined Grains White breads White rice Biscuits Cakes Added sugars Soda Sweetened Coffee and tea Fruit drink and lemonade Many breakfast cereals Granola bars Deserts and candy
Fats	Unsaturated Fats Vegetable oils Nuts, nut butters Fish Avocado	Saturated fats Butter and stick margarine Heavy Cream Cream cheese
Protein	Lean Meats Poultry Eggs Beans, peas, lentils Seafood Soy products Nuts and Seeds	Fatty meats Beef ribs Sausage Processed meats Fried meats
Fruits	Whole Fruits Berries Melons Citrus Fruits (oranges, grapefruit, limes) Other fruits (apples, banana, pears, apricot, etc.)	Juices that are not 100% fruit juice
Vegetables	Vegetables of all types including Dark green vegetables (spinach, kale, broccoli) Red and Orange vegetables (peppers, squash, carrots) Starchy vegetables (potatoes, corn, yucca, jicama) Beans, peas, lentils Other vegetables (sprouts, cauliflower, asparagus, eggplant, etc.)	Fried vegetables
Dairy	Fat-free low-fat dairy Skim milk 1% (low fat) milk Low-fat or fat-free yogurt Cheeses Non-dairy alternatives such as soy milk	Full-fat dairy: Whole milk Full-fat yogurt Full-fat cheeses
Additional resources on following for healthful diet plan: www.choosemyplate.gov (accessed on 14 December 2021) www.dietaryguidelines.gov (accessed on 14 December 2021)		

Adapted from data found in Academy of Nutrition and Dietetics CF Nutrition Guidelines and the Dietary Guidelines for Americans.
